# Management of Hypertension in the Elderly Patient at Abidjan Cardiology Institute (Ivory Coast)

**DOI:** 10.1155/2012/651634

**Published:** 2011-10-18

**Authors:** K. E. Kramoh, E. Aké-Traboulsi, C. Konin, Y. N'goran, I. Coulibaly, A. Adoubi, J. Koffi, J. B. Anzouan-Kacou, M. Guikahue

**Affiliations:** Institute of Cardiology of Abidjan (Ivory Coast), BPV 206, Abidjan, Cote d'Ivoire

## Abstract

*Background*. Since the treatment of hypertension is beneficial for the elderly, we have undertaken this study that aims to evaluate the management of hypertension in elderly patient in Côte d'Ivoire. *Methods*. A retrospective study was conducted among 854 hypertensive elderly patients of Abidjan Cardiology Institute who were followed for a minimum of one year, between January 2000 and December 2009. *Results*. The patients mean age was 73.1 ± 5.3 years, and 59% were women. At the first presentation, it was mostly systolic-diastolic hypertension (51.8%) and isolated systolic hypertension (38.5%). Mean blood pressure was 169.4 ± 28.4 mmHg for systolic, 95.3 ± 15.7 mmHg for diastolic, and 74.1 ± 22.8 mmHg for pulse pressure. Pulse pressure was ≥60 mmHg in 80.4%. According to the European Guidelines stratification of the cardiovascular risk-excess attributable to high blood pressure, 82.1% of the sample had a very high added risk. The pharmacological therapy was prescribed in 93.5%. More than 66% of patients were receiving ≥2 antihypertensive drugs including fixed-dose combination drugs. The most common agents used were diuretics (63.5%) followed by angiotensin-converting enzyme inhibitors or angiotensin receptor blockers in 61.3%. The most common agents used for monotherapy were calcium antagonists. When ≥2 drugs were used, diuretics and angiotensin-converting enzyme inhibitors or angiotensin receptor blockers were the most common. Blood pressure control was achieved in 42.6%. *Conclusion*. The control of elderly hypertension can be effective in Sub-Saharan Africa. He required at least two antihypertensive drugs to meet the recommended blood pressure target.

## 1. Introduction

Hypertension is an important worldwide public-health challenge because it is one of the most common chronic conditions [1, 2]. The prevalence of hypertension in Sub-Saharan Africa is between 12.5 and 26.9% [3]. In 2005 the World Health Organization stepwise approach to surveillance of noncommunicable diseases risk factors established prevalence of hypertension in Côte d'Ivoire to 21.7% [4].

Hypertension is a major risk factor for cardiovascular (CV) disease [5–7]. It remains an important cause of coronary heart disease, cerebrovascular disease, peripheral artery disease, and heart failure [8]. 

Age is the most powerful risk factor for hypertension, death, and cardiovascular death [9]. The worldwide increase in the elderly population (age ≥ 65 years) is associated with concurrent increases in prevalence of systemic hypertension and morbidity and mortality from vascular complications of hypertensive disease [10].

Recently, numerous large clinical trials have provided evidence of the benefits of reducing BP in the elderly. Meta-analysis of clinical trials showed that treatment of hypertension in older adults is as beneficial as that in younger adults [11, 12]. It is well established now that the treatment of hypertension in elderly patient was associated with a reduction in the rate of fatal or nonfatal stroke, a reduction in the rate of death from stroke, a reduction in the rate of death from any cause, a reduction in the rate of death from cardiovascular causes, and a reduction in the rate of heart failure [12, 13].

There are few Sub-Saharan African data about management of hypertension in elderly. It was in this context that we undertook the present study at Institute of Cardiology of Abidjan, the single university hospital managing cardiovascular diseases in Côte d'Ivoire. This study aims to describe characteristics, risk factors, treatment and control of blood pressure of elderly hypertensive patients.

## 2. Methods

We undertook a retrospective descriptive study involving patients seen at the outpatient clinics of the Institute of Cardiology of Abidjan. The study period spans 10 years, between January 2000 and December 2009. The study population was hypertensive elders (aged at least 65 years) with a regular followup at the Institute of Cardiology of Abidjan within one year. This series includes patients who had been on an initial treatment upon referral to our center. Data were collected from the information contained in medical records. 

We used the definition and classification hypertension standards of the European Society of Hypertension (ESH) and the European Society of Cardiology (ESC) [7]. We have separated the systolic-diastolic hypertension from isolated systolic hypertension and from isolated diastolic hypertension. Pulse pressure has been considered pathological when it was ≥60 mmHg. The data collected were age, sex, blood pressure at initial presentation and during the followup (one month, 2 months, 3 months, 6 months, 1 year or last presentation), the coexistence of other cardiovascular risk factors, the impact of hypertension, and the treatment modalities. The following additional CV risk factors were document when present: current smoking, dyslipidaemia, diabetes, and obesity. Obesity was defined by a body mass index over 30 kg/m^2^. Waist circumference was recorded when available. Dyslipidaemia was defined according to our biochemistry laboratory standard when the level of total cholesterol was >2 g/dL, LDL-cholesterol >1.4 g/dL, or HDL-cholesterol <0.4 g/L. Diabetic patients were informed and treated for diabetes as per standard clinical practice. New diabetic patients detected at Institute of Cardiology of Abidjan were diagnosed based on the standard value in our laboratory, fasting plasma glucose >1.26 g/dL on repeated measurement. The overall management of diabetes was coordinated by the individual patient's physician and not by the Cardiology staff of Institute of Cardiology of Abidjan. The assessment of the impact of hypertension has systematically included electrocardiogram and plasma creatinine. All strokes were documented by a brain computerized tomography scan imaging. Echocardiogram was frequently performed, and the results were included in the data collection. Finally, when other tests were seldom performed (e.g., albuminuria, microalbuminuria, the fundscopy, and 24-hour ambulatory blood pressure), they were not used for analysis. 

The ESH and ESC categorization of total risk as low, moderate, high, and very high added risk has the merit of simplicity and was, therefore, chosen for risk stratification [7]. In addition, we focused on the evolution of blood pressure during followup. Blood pressure control was defined as a treated blood pressure <140 mmHg systolic and <90 mmHg diastolic and was ascertained by direct measurement of blood pressure.

Data analysis was conducted using IBM SPSS Statistics 17 software. Univariate analysis was performed for significant associations. A *P *value of ≤ 0.05 was considered for statistical significance. 

## 3. Results

During the study period, 2575 hypertensive Black Africans patients have had a regular follow-up of at least one year. Among these patients, 849 were elderly subjects. The mean age was 73.1 ± 5.3 years (range 65–98 years), 59% were female. At first presentation blood pressure was inappropriately controlled in 90.8% patients. 

It was mostly systolic-diastolic hypertension (51.8%) and isolated systolic hypertension (38.5%). Diastolic hypertension was observed in 0.5% (Table 1). Mean blood pressure was 169.4 ± 28.4 mmHg for systolic, 95.3 ± 15.7 mmHg for diastolic, and 74.1 ± 22.8 mmHg for pulse pressure. Blood pressure of men was not significantly different from that of women (Table 2). Pulse pressure was ≥60 mmHg in 80.4%. Overall, patients were diagnosed with hypertension for an average of 4 ± 6.7 years (range 1–47) at the time of their initial assessment in our clinics. The mean follow-up duration in ICA was 3 ± 1.8 years (range 1 to 18 years). 

Diabetes was found in 18.6% of patients. It was type 2 in 61.4%. At least one target organ damage was observed in 50%. Isolated cardiac complications were the most frequent (37.9%). The cardiovascular risk factors other than hypertension and the results of target organ damage are reported in Table 1. Habitual alcohol consume was found in 28.4%. 

Echocardiography was performed in 50.2%. The recorded anomalies were a left ventricular diastolic dysfunction (39.2%), hypertensive cardiomyopathy (32.1%) and minor lesions (slight valve regurgitation, valvular sclerosis, valvular calcification) (19.6%). 

Application of the cardiovascular risk stratification according to the European Guideline for the management of arterial hypertension identified a very high added risk in 82.1%, a high added risk in 4.8%, a moderate add risk in 7.5% and a low add risk in 5.7%. 

In addition to lifestyle changes, 93.5% of patients received antihypertensive drugs (Table 2). Of those on treatment for high blood pressure, the most common agents used were diuretics in 63.5%, followed by blockers of the renin-angiotensin system (RAS) in 61.3% (either angiotensin converting enzyme inhibitors or angiotensin receptor blockers), calcium antagonists (31.6%), *β*-blockers (19%), and centrally acting sympatholytics (4.5%). Antihypertensive drugs were used in monotherapy or combination (Table 3). As a monotherapy, the most prescribed drug class was calcium antagonists (36.3%), followed by RAS blocker (32%). Polytherapy (more than 2 antihypertensive drugs including fixed-dose combination drugs) was used for 67% of patients on treatment for hypertension. The most common combination of drugs among those taking 2 agents was RAS blockers plus diuretics (69.8%). 

Diabetic patients as well as those with kidney failure have received significantly more RAS-blockers for the treatment of hypertension than other patients (*P* = 0.03 for diabetic and *P* = 0.04 for renal failure). 

Between the first and the last visit, blood pressure decreased significantly (Table 3). It was for systolic blood pressure by 15.9 mmHg, diastolic blood pressure by 8.2 mmHg, and pulse pressure by 7.6 mmHg. Hypertension was controlled in 42.6% of patients. 

**Figure 1:**
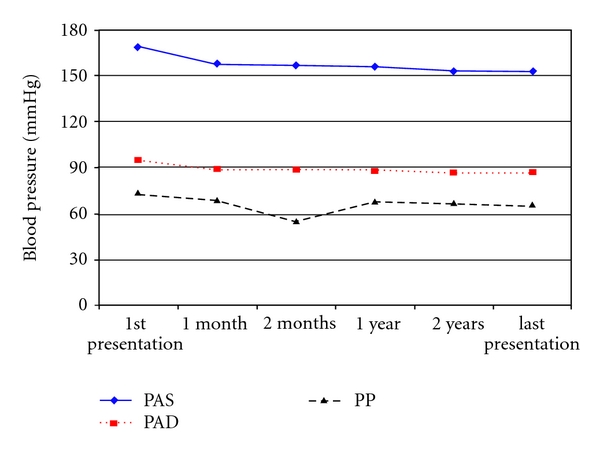
Changes in blood pressure during followup.

Figure 1 shows changes in blood pressure during followup. The sex (*P* = 0.88), age (*P* = 0.48), duration of treatment of hypertension (*P* = 0.13), the number of consultation (*P* = 0.13), and the duration of hypertension (*P* = 0.27) did not influence the control of blood pressure. Hypertension was best controlled with multiple drugs therapy (1.7 drugs for controlled patients as compared to 1.4 for to uncontrolled patients, *P* < 0.001). Pulse pressure was significantly lowered in females (*P* = 0.03) (Table 4).

## 4. Discussion

This study found that a significant proportion of patients (33%) who visit the ICA are elderly. In the elderly a significant part of hypertension is represented by systolic hypertension. Increased arterial stiffness may increase cardiovascular morbidity and mortality because of an elevation of systolic blood pressure (SBP), which raises left ventricular afterload, and because of a decrease in diastolic blood pressure (DBP), which alters coronary perfusion [14]. These elderly patients have a high pulse pressure (PP). Elevated PP is a powerful independent predictor of cardiovascular end points in the elderly [15–18]. 

According to Skurnick et al. [19], the PP levels of women were lower than those of men in early adulthood and higher in older ages. But in our study, although the difference was not statistically significant, the PP of men was higher than women's PP. Moreover, under hypertension treatment, the PP of women had significantly regressed compared to men's.

Cardiovascular risk of our patients was very high in most cases. As has been previously described, it is suggested that Black Africans present more severe forms of arterial hypertension and a greater risk of target organ damage [20–23]. Overall, uncontrolled blood pressure remains the main factor for target organ damage more frequently in Sub-Saharan Africa compared to western countries [24]. 

The main benefits of antihypertensive treatment are blood pressure lowering per se, largely independent of the drugs employed. Diuretics, *β*-blockers, calcium antagonists, and RAS blockers can adequately lower blood pressure, significantly improving cardiovascular outcome [25]. Several properties of the thiazide-type diuretics have led to them being recommended as first-line therapy in older adults with uncomplicated stage 1 hypertension. At low doses (<25 mg/day of hydrochlorothiazide or equivalent), these agents have been demonstrated in randomized controlled trials to reduce mortality, stroke, and cardiovascular events in the older hypertensive population [26]. There is good synergy with agents of different classes (RAS-blockers and calcium antagonists) and most importantly in the elderly; these drugs preferentially lower SBP relative to DBP. In our study, diuretics have been widely prescribed. In our environment, the added benefit to of diuretics is their low cost. RAS-blockers were also widely prescribed because of comorbidities such as diabetes, left ventricle hypertrophy or kidney failure, situations that required a preferential indication of RAS-blockers. Furthermore, HYVET [12] recommended the addition of a RAS-blocker in the event of insufficient control of BP. The RAS-blocker diuretic combination was by far the most used in our study. 

In monotherapy, calcium antagonists were the most prescribed. Calcium antagonists have shown effectiveness in lowering BP in the older hypertensive patient. Significant reductions in stroke risk in older hypertensive patients were demonstrated in the Systolic Hypertension Europe and China Trials [27, 28]. Furthermore, results from patients with very high cardiac risk enrolled in ACCOMPLISH trial demonstrated the superiority of an ACEI-calcium antagonists (amlodipine) combination over an ACE-thiazide combination with regard to a decrease in cardiovascular events despite comparable BP-lowering effects [29]. 

In most cases, combination therapy was required for our patients. According to Aronow [30] if blood pressure is more than 20/10 mmHg above the target BP, treatment should be initiated with two antihypertensive drugs. In our study, hypertension was best controlled with multiple drugs therapy. One should certainly not hesitate to use more than one antihypertensive drug even in elderly patients if the target blood pressure is not reached. Particular attention must be given to eventual side-effects in elderly population. Control rate of hypertension (42.6%) was acceptable in our Sub-Saharan African context. 

## 5. Study Limitation

There is no information on treatment tolerance, particularly on orthostatic hypotension occurrence in the elderly often polymedicated patients. This limit is due to the retrospective nature of our study. Also in these elderly patients with high cardiovascular risk, it would be interesting to describe cardiovascular events occurred during the year-long or more followup. Furthermore, the control rate of hypertension obtained does not reflect the reality of the management of hypertension in Côte d'Ivoire. There is certainly a bias due to the method of recruitment of our population. Patients who were regularly monitored for at least one year showed probably best treatment adherence.

## 6. Conclusion

Despite the classical reduced life expectancy in Sub-Saharan African population because of various illnesses, numerous elderly people exist. One must deal with the specific health needs of the elderly. Their blood pressure is characterized by the frequency of isolated systolic hypertension. Elderly hypertensive patients have a very high cardiovascular risk. Diuretics as recommended were the drugs most prescribed. The control rate of hypertension was significant, mostly at the cost of combination therapy.

## Figures and Tables

**Table 1:** Population distribution according to the classification of blood pressure, the other cardiovascular risk factor, and target organ damage at first presentation.

Clinical data	%
Classification of blood pressure	

Controlled blood pressure	9.2
Systolic and diastolic hypertension (51.8%)	
Grade1 hypertension	4.8
Grade 2 hypertension	14.7
Grade 3 hypertension	32.3
Isolated systolic hypertension (38.5%)	
Grade1 hypertension	16.8
Grade 2 hypertension	12.7
Grade 3 hypertension	9
Isolated diastolic hypertension	0.5

Other cardiovascular risk factor	

Dyslipidemia	56%
Diabetes	18.6
Smoking	23.7
Obesity	33.8
Abnormal pulse pressure	80.4

Organ damage	

No organ damage	36.3
One organ damage	
Cardiovascular	37.9
Neurological	9.8
Renal	2.7
More than one organ damage	
Cardiovascular + renal	8.5
Cardiovascular + neurological	3.1
Renal + neurological	0.6
Cardiovascular + neurological + renal	1.1

**Table 2:** Type of drugs used.

Type of drug	**%**
*One drug* *(33.1%) *	
Calcium antagonists	36.3
RAS blockers	32
Diuretics	16
*β*-blockers	10.7
CAS	5

*Two drugs* *(53.8%) *	
RAS blockers + Diuretics	69.8
*β*-blockers + Diuretics	8.5
*β*-blockers + Calcium antagonists	7.4
Diuretics + Calcium antagonist	5.5
RAS Blockers + Calcium antagonists	3.5
RAS Blockers + *β*-blockers	3.5
CAS + (diuretics or RAS blockers or calcium antagonist or *β*-blockers)	1.8

*Three drugs (11%)*	
RAS blockers + Diuretics + Calcium antagonists	51.7
RAS blockers + Diuretics + *β*-blockers	18.4
Diuretics + *β*-blockers + Calcium antagonists	17.2
RAS blockers + *β*-blockers + Calcium antagonists	2.3
Other using CAS	10.4

*Four drugs* *(2.2%) *	
RAS blockers + Diuretics + Calcium antagonists + *β*-blockers	58.8
RAS blockers + Diuretics + Calcium antagonists+ CAS	35.3
RAS blockers + Diuretics + *β*-blockers+ CAS	5.9

RAS blockers: angiotensin-converting enzyme inhibitors or angiotensin receptor blockers, CAS: Centrally acting sympatholytics.

**Table 3:** Change in blood pressure for all patients.

Blood pressure (mmHg)	First presentation	Last presentation	*P*
SBP	169.4 ± 28.4	153.5 ± 26.1	<0.001
DBP	95.3 ± 15.7	87.1 ± 14.3	<0.001
PP	74.1 ± 22.7	66.5 ± 19.9	<0.001

**Table 4:** Change in blood pressure by gender.

	Blood pressure (mmHg)	Male	Female	*P*
	SBP	171 ± 28.6	168.3 ± 28.3	0.28
First presentation	DBP	95 ± 16.3	95.4 ± 15.3	0.39
	PP	76 ± 23.3	72.9 ± 22.3	0.24

	SBP	155.4 ± 27.1	152.2 ± 25.4	0.88
Last presentation	DBP	87.9 ± 14.9	86.5 ± 13.9	0.89
	PP	67.4 ± 19.5	65.7 ± 20.2	0.03

Control rate		39.2	45.0	0.88

DBP: Diastolic blood pressure, PP: Pulse pressure, SBP: Systolic blood pressure.
